# Establishment of lactate-metabolism-related signature to predict prognosis and immunotherapy response in patients with colon adenocarcinoma

**DOI:** 10.3389/fonc.2022.958221

**Published:** 2022-09-14

**Authors:** Zhengrong Zou, Yongjie Chai, Qi Li, Xuan Lin, Qingfang He, Qiusheng Xiong

**Affiliations:** ^1^ Department of Emergency Trauma Center, The First Affiliated Hospital of Gannan Medical University, Ganzhou, China; ^2^ Department of Anorectal Surgery, Zhucheng People’s Hospital, Zhucheng, China; ^3^ Basic Medicine Department of Chuxiong Medical and Pharmaceutical College, Chuxiong, China; ^4^ Department of General Surgery, The First Affiliated Hospital of Gannan Medical University, Ganzhou, China

**Keywords:** colon adenocarcinoma, lactate metabolism, risk signature, tumor immune microenvironment, immunotherapy

## Abstract

The outcome of colon adenocarcinoma (COAD) patients remains dismal, and lactate metabolism has been characterized to promote tumor development and immune evasion. Based on the above background, it is worthwhile to explore novel prognostic and therapeutic biomarkers for COAD patients from the aspect of lactate metabolism. Above all, 228 available lactate-metabolism-related genes (LMRGs) were acquired, and the landscape of copy number variation and the expression difference of mRNA levels between colon normal and tumor samples were investigated among these LMRGs. Importantly, eight overall survival (OS)-involved LMRGs were then distinguished by means of univariate Cox regression analysis in both GSE40967 and TCGA-COAD data sets. Subsequently, prognostic risk scores were established, integrating seven OS-related LMRGs by LASSO Cox regression analysis in the GSE40967 set, and then verified in the TCGA-COAD cohort. From the comprehensive analyses, COAD patients with high risk had comparatively more inferior survival probability in all populations of the study, and they tended to have more severe clinicopathological features with the risk score increasing. Moreover, by integrating age, AJCC T and pathological stage, and risk score, we constructed a prognostic nomogram that demonstrated great prediction effectiveness for OS of COAD patients. Furthermore, the potential effect of various risk score on tumor immune was assessed from enrichment of immune-related pathways, tumor-infiltrating immune cells, and expression levels of immune checkpoints separately. We could draw a conclusion that COAD patients with higher lactate-metabolism-related risk scores may acquire an immunosuppressive tumor microenvironment, which subsequently led to immune escapes and poor prognoses. Conclusively, all findings in the present study illustrate a great prognostic value of the lactate-metabolism-related risk signature, providing more in-depth insights into the indispensable function of lactate metabolism in prognosis and tumor immunity of COAD.

## Introduction

Colon adenocarcinoma (COAD) has been the third most common malignant tumor with 10% of all cancers and high mortality (1). Therapeutic advancements, including surgical technique, chemotherapy, and molecular targeted therapies, have greatly improved the outcome of patients with COAD (2). However, with the increases in incidence and drug resistance, the prognosis of some patients with COAD remains dismal (3). To further enhance the curative effect and survival of such population, more effective biomarkers and more accurate cancer identification are still urgent and worth exploring.

To date, there has been a general consensus that aerobic glycolysis (the Warburg effect) has emerged as a metabolic hallmark of cancers, and tumor cells secrete large amounts of lactate, which always results in lactate accumulation in the tumor microenvironment (TME) (4). Hence, lactate metabolism has attracted more attention in cancer metabolic research recently. A considerable amount of evidence has uncovered the nonnegligible role of lactate metabolism alterations as biomarkers of cancer prognoses (5–8). For COAD, lactate originated by the noncancer stem cells promoted self-renewal of cancer stem cells (CSCs) and consequently contributed to tumor progression (9). Besides this, meta-analyses revealed that high levels of lactate dehydrogenase were correlated with unfavorable overall survival (OS) in colorectal cancer (CRC) patients (10). Nonetheless, a full-scale landscape of the impact of lactate metabolism on prognosis of COAD still lacks.

COAD development is verified to be a complicated process involving the interactions between the tumor, TME, and host immune system (11). Accumulating evidence reveals that TME is closely related to the progression, relapse, metastasis, and therapeutic resistance of CRC (12). To be exact, the TME is especially lactate-enriched (13). For tumor-infiltrating immune cells, which are a part of the complex microenvironment, playing a leading role in the TME, lactate accumulation supported tumor immune escape by depressing the cytotoxic activities of T cells and connatural lymphocytes such as natural killer (NK) and natural killer T (NKT) cells (14, 15). In CRC, lactate-mediated acidification of TME is revealed to induce apoptosis of liver-resident NK cells in liver metastasis (16). In addition, decreasing lactate production in cancer cells is observed to synergize with immunotherapy by preventing the acidification of the TME in melanoma (17). On the whole, the effect of lactate metabolism on the TME cannot be underestimated. However, a comprehensive analysis of such influence in COAD has not yet been reached.

In this study, we identified prognostic lactate-metabolism-related genes (LMRGs) and establish a reliable nomogram model on the basis of LMRGs to predict the survival outcomes of COAD patients. Moreover, the potential relationship between the signature and the TME was further explored. Our study provides more evidence that lactate metabolism is strongly correlated with patient prognosis and tumor immunity in COAD.

## Materials and methods

### Data retrieval and collection of LMRGs

From The Cancer Genome Atlas (TCGA) database (41 normal colon samples and 473 COAD samples, https://portal.gdc.cancer.gov/repository) and GSE40967 in the Gene Expression Omnibus (GEO) database (585 COAD samples, https://www.ncbi.nlm.nih.gov/geo/), the public transcriptome expression matrices and clinical information of COAD patients were retrieved. Afterward, 573 COAD patients in GSE40967 were set to the training set, whereas 457 COAD samples of TCGA were selected as an external validation set after exclusion of patients with no OS information. From the Molecular Signature Database v7.5.1 (MSigDB), 284 LMRGs were downloaded (18). Furthermore, 228 overlapping LMRGs were collected for ulterior analyses after intersecting the aforementioned 284 LMRGs with all genes in GSE40967 and TCGA-COAD data sets (**Supplementary Figure 1**).

### Verification of copy number variation (CNV) frequency and differentially expressed genes (DEGs) among LMRGs

The CNV data of patients from TCGA-COAD was attained in the UCSC Xena database (https://xenabrowser.net/datapages/). Subsequently, the CNV frequency of the above 228 LMRGs was computed, and a bidirectional column chart was used to visualize the result. The DEGs within LMRGs were verified after comparing the normal and cancer samples in the TCGA-COAD data set when the threshold was set with false discovery rate (FDR)< 0.05 and |log2FC| > 1 using the “edgR” R package (19, 20). These significant DEGs were also described with a heat map and a volcano plot.

### Acquisition of OS-related LMRGs in COAD

To demonstrate the profound prognostic significance of 228 LMRGs in COAD, Cox proportional hazards regression analyses were carried out for univariate analyses to obtain OS-related LMRGs with *P*<.05 in the GSE40967 (*n* = 573) and TCGA-COAD (*n* = 457) cohorts, respectively. Ulteriorly, the overlapping OS-related LMRGs were screened out for further research. Meanwhile, the relevant characteristics among the above LMRGs were illustrated in a correlation matrix plot, and the “RCircos” R package was utilized to display mRNA expressions and chromosomal positions of those candidate LMRGs (21).

### Establishment and validation of lactate metabolism–correlated prognostic signature in COAD

To establish a statistically prognostic signature according to these eligible OS-related LMRGs, least absolute shrinkage and selection operator (LASSO) Cox regression analysis was accomplished in the training cohort. In addition, seven LMRGs were retrieved to construct a signature for COAD patients, whereas the prognostic significance of each LMRG included in the signature was portrayed, respectively. According to the predictive signature, the lactate-metabolism-related risk score of individual COAD patient would be calculated as follows:


Risk   Score=∑Expression   of  Each  LMRG∗  Corresponding  Regression  Coefficient

Meanwhile, the risk score was adjusted by a linear transformation in every data set with the following formula:


$$adj.Risk\;score=\frac{\text{Risk score}-\text{min}\left(\text{Risk score}\right)}{\text{max}\left(\text{Risk score}\right)\;-\text{min}\left(\text{Risk score}\right)\;}$$

Subsequently, patients in each cohort were divided into high- and low-risk subgroups using the median cutoff value. To uncover the feasibility of the risk model, Kaplan–Meier survival analysis of OS difference was executed between high- and low-risk groups in two data sets separately, and Kaplan–Meier survival analyses of progression-free survival (PFS), disease-free survival (DFS), and disease-specific survival (DSS) were further performed between different risk groups in the TCGA-COAD cohort.

### Completed investigation of risk score and clinical parameters in patients with COAD

To elucidate the availability of the risk signature based on LMRGs in clinical situations, we compared the distribution of adjusted risk values with different degrees of various clinicopathologic parameters using boxplots with the Kruskal test. Additionally, heat maps were plotted to decipher the correlation between each selected LMRG’s expressions and important clinical indicators, comprising risk score, T, N, AJCC stage, and survival status in the training and validation sets.

### Construction and evaluation of lactate-metabolism-related clinical nomogram in COAD

Furthermore, univariate and multivariate Cox regression analyses were delineated to explore whether the lactate-metabolism-related risk score could be an independent predictor of COAD. Based on the results presented, a lactate-metabolism-related nomogram, integrating risk score, age, T, and AJCC stage in the GSE40967 was constructed through the “rms” and “regplot” R packages (22, 23). In an effort to evaluate the predictive performance of the nomogram, we performed the calibration analysis and decision curve analysis (DCA) and plotted corresponding curves (24).

### Identification of different biological functions within two risk subgroups

The “GSVA” R package was carried out to investigate the distinctions of biological processes and signaling pathways between high- and low-risk groups in the training and validation sets, respectively (25, 26). “c2.cp.kegg.v7.5.1.symbols.gmt” [KEGG] was dug out from MSigDB as the reference molecular signature database, and *P* values<.05 were deemed statistically significant after being adjusted. Ultimately, the top 20 significant KEGG pathways were displayed as heat maps.

### Potential implications for TME landscape and immunotherapy based on the risk signature

To reveal the potential implications for immunotherapy based on lactate-metabolism-related risk score, different expression levels of three immunologic checkpoints, namely PDCD1 (PD-1), CD274 (PD-L1), and CTLA4, were detected between high- and low-risk groups using the Wilcox test. To determine the relative tumor-infiltrating abundance of 22 immune cells in the two subgroups, the CIBERSORT deconvolution algorithm was employed in the training set (27, 28).

### Statistical analysis

Statistical analyses were done with R software (Version 4.0.2, http://www.R-project.org). The log-rank test was applied to conduct the comparison between Kaplan–Meier curves in this study (29). The Kruskal test was utilized to uncover the differences of adjusted risk scores in various clinical parameters. The discrepancy of checkpoints in low- and high-risk groups was detected by the Wilcox test. The correlation matrix diagram was examined using Spearman’s correlation test. All *P* values were bilateral, and statistical significances were set at *P*<.05.

## Results

### Identification of prognostic LMRGs in COAD

**Figure 1:**
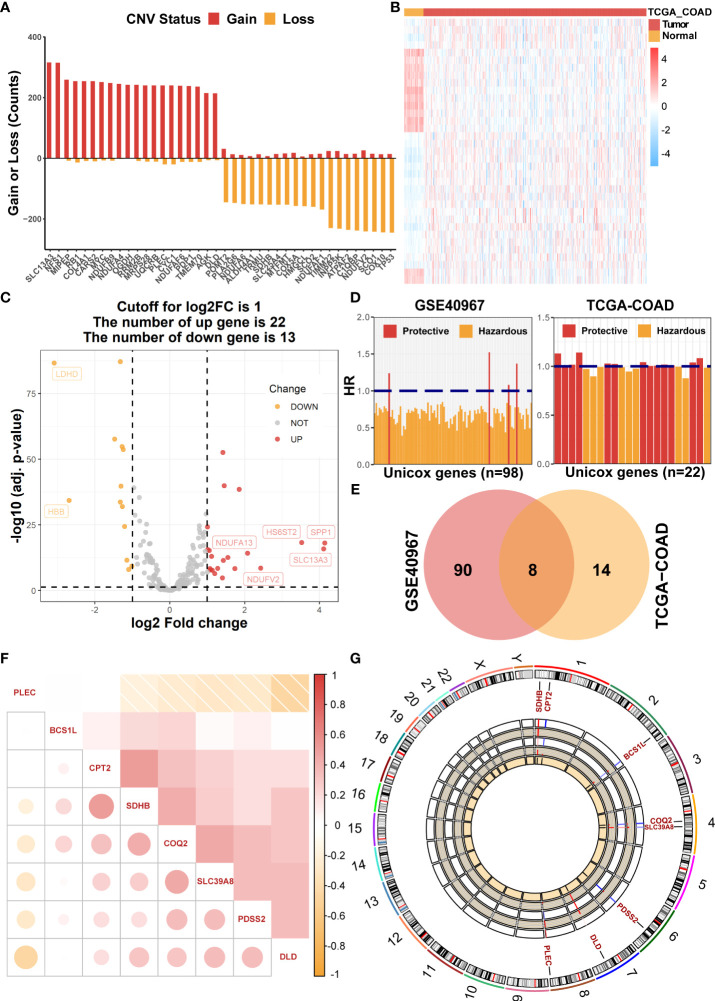
Identification of prognostic LMRGs in COAD patients. **(A)** The global CNV frequency of LMRGs in the TCGA-COAD cohort. **(B)** The heat map of differentially expressed LMRGs between adjacent and tumor samples in TCGA-COAD. **(C)** The volcano plot presenting differentially expressed genes among LMRGs. **(D)** OS-related LMRGs in GSE40967 and TCGA-COAD cohorts, respectively. **(E)** The Venn diagram to identify eight intersected prognostic LMRGs. **(F)** The correlation matrix plot exhibiting eight candidate LMRGs. **(G)** The Circos plot to illustrate regions on chromosomes and expressions of eight candidate LMRGs.

Initially, 228 common LMRGs were obtained through the intersection of two databases (GSE40967 and TCGA-COAD). We first assessed the global CNV alterations of 228 LMRGs in TCGA-COAD, which showed that there existed extensive CNV mutations among LMRGs. The top 20 genes in CNV amplification and deletion status are displayed in **Figure 1A** together. Then, the mRNA expression profiles of COAD samples in TCGA-COAD were analyzed to find differentially expressed LMRGs with the threshold of FDR< 0.05, |log2FC| > 1 and visualization of a heat map (**Figure 1B**) and a volcano plot (**Figure 1C**), respectively. Moreover, to identify prognostic candidate LMRGs in COAD for further research, univariate Cox regression analyses were performed to sift OS-related LMRGs in both TCGA-COAD and GSE40967 data sets (**Figure 1D**). In total, 22 and 98 significant OS-related genes were yielded, respectively, and eight equitant LMRGs (PLEC, BCS1L, CPT2, SDHB, COQ2, SLC39A8, PDSS2, and DLD) were factored into subsequent analyses (**Figure 1E**). Meanwhile, to obtain a more advanced understanding of these genes, a correlation network plot was presented to unravel the correlation features among eight eligible LMRGs (**Figure 1F**), and chromosomal positions and mRNA expression levels of these eight genes were illustrated by a circos plot (**Figure 1G**).

### Establishment and validation of lactate-metabolism-related prognostic signature for patients with COAD

**Figure 2:**
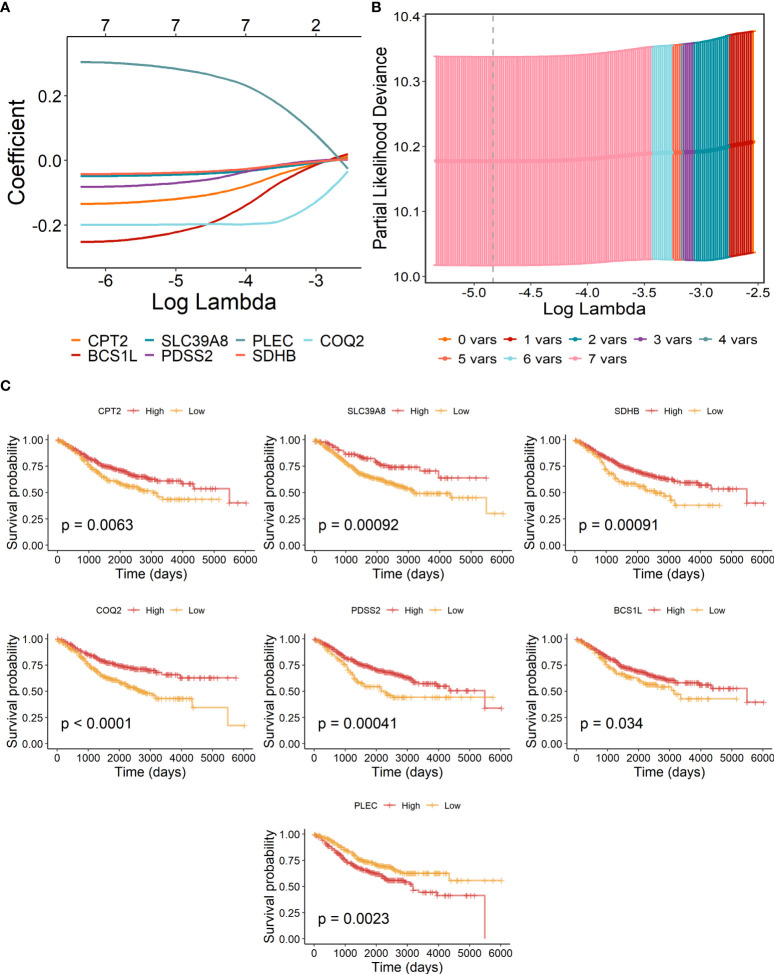
Construction of lactate-metabolism-related prognostic signature for COAD patients of the GSE40967 training group. **(A)** LASSO Cox regression analysis of the eight prognostic LMRGs. **(B)** Partial likelihood deviance for the LASSO regression to determine seven optimal prognostic LMRGs. **(C)** KM survival analyses of OS on the basis of mRNA expression levels of 7 LMRGs.

The above eight candidate LMRGs were subsequently analyzed through carrying out the LASSO Cox regression analysis in COAD patients of the GSE40967 training data set, and seven pivotal genes (PLEC, BCS1L, CPT2, SDHB, COQ2, SLC39A8, and PDSS2) were determined to build the prognostic signature (**Figures 2A, B**). In addition, Kaplan–Meier survival analyses were conducted to investigate the survival capability of every signature-contained gene in the training (**Figure 2C**) data set. From the results, we found that high expression of PLEC and low expressions of BCS1L, CPT2, SDHB, COQ2, SLC39A8, and PDSS2 were significantly correlated with more unfavorable OS in COAD, which further supports the effectiveness of the selected genes. At length, a risk signature was constructed as follows: risk score = Expression of PLEC * 0.265886 - Expression of CPT2 * 0.106776 - Expression of BCS1L * 0.196516 - Expression of SLC39A8 * 0.040630 - Expression of PDSS2 * 0.059233 - Expression of SDHB * 0.033911- Expression of COQ2 * 0.196516.

**Figure 3:**
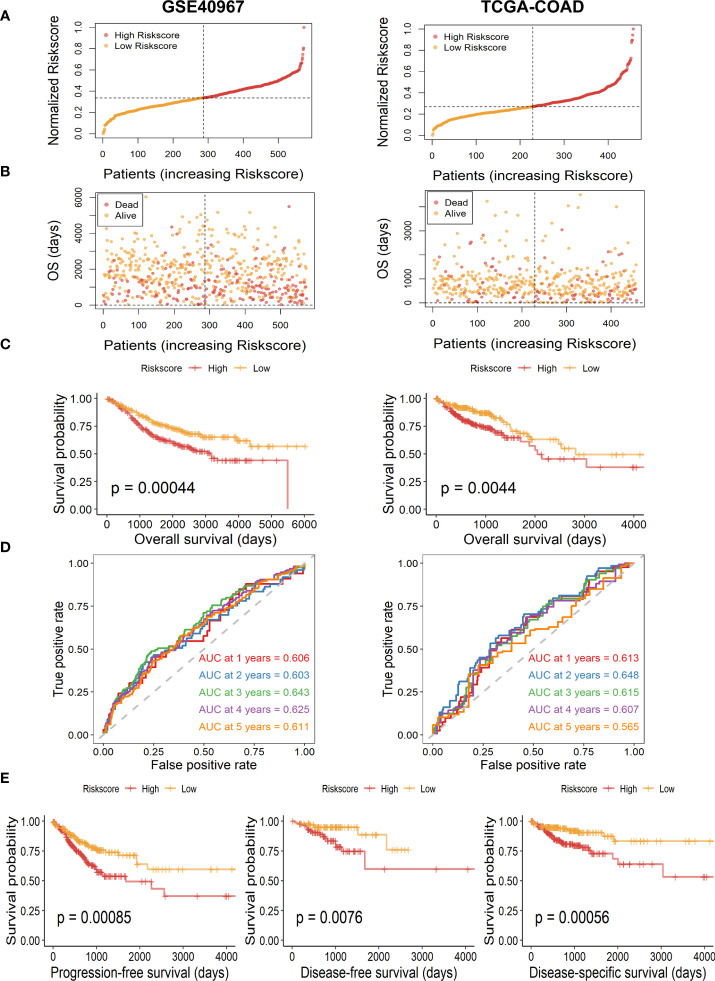
Assessment and validation of the availability of risk score in GSE40967 and TCGA-COAD sets. **(A)** Risk score distribution of COAD samples with different risks. **(B)** The distribution of risk score and OS time of COAD patients. **(C)** The Kaplan–Meier survival curves of OS according to high- and low-risk groups in patients with COAD. **(D)** The time-dependent ROC curves and AUC values for OS of the prognostic risk model. **(E)** The Kaplan–Meier survival curves of PFS, DFS, and DSS according to different risk groups.

Then, to further test the prognostic value of our risk signature, patients of the TCGA-COAD training set and the GSE40967 validation set were independently segregated into high- and low-risk subgroups based on the median value of the risk score (**Figure 3A**). As we expected, patients of the high-risk group had higher incidence of deaths and shorter OS probability in both training and validation cohorts (**Figure 3B**). Meanwhile, the Kaplan–Meier survival analysis of OS indicated that high-risk COAD patients had considerably more unfavorable OS probability across all cohorts (**Figure 3C**, GSE40967, *p* = .00044 TCGA-COAD, *p* = .0044). Moreover, we performed survival analysis in patients of TCGA-COAD according to another survival index, and the results show that patients with high risk had similarly poorer PFS (*p* = .00085), DFS (*p* = .0076), and DSS (*p* = .00056), which strongly proves the above conclusions (**Figure 3E**). Additionally, we carried out time-dependent ROC curves to evaluate the performance of the risk prediction model, which depicted good predictive capability with AUCs in the training set as 0.606, 0.603, 0.643, 0.625, and 0.611 for 1-, 2-, 3-, 4-, and 5-year OS, and AUCs in the validation set were 0.613, 0.648, 0.615, 0.607, and 0.565 for 1-, 2-, 3-, 4-, and 5-year OS, respectively (**Figure 3D**).

### Evaluation of the correlation between risk scores and clinicopathological indicators in COAD

**Figure 4:**
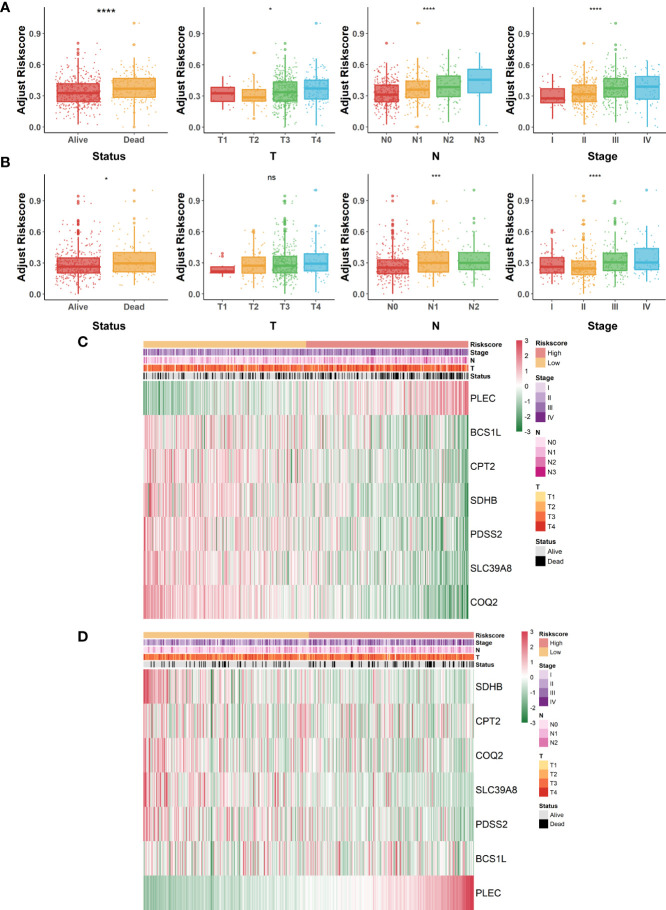
Correlation of lactate-metabolism-related risk signature and clinical indicators in COAD patients. **(A, B)** Box plots to present the relationship between different risk groups and clinicopathological characteristics of patients in GSE40967 **(A)** and TCGA-COAD **(B)**, respectively. **(C, D)** Correlation heat maps of risk signature-contained LMRGs and clinicopathological factors in GSE40967 **(C)** and TCGA-COAD **(D)** separately. *p < 0.05; ***p < 0.001; ****p < 0.0001; ns, not significant.

Overall appraisal of the relationship between risk scores and common clinicopathological factors in COAD patients was then assessed. In the GSE40967 cohort, conspicuous discrepancies were observed between risk scores and clinical features, including survival status (*p*<.0001), T (*p*<.05), N (*p*<.0001), and AJCC stage (*p*<.0001) (**Figure 4A**). Similarly, significant correlations were also observed in the above indicators of the TCGA-COAD cohort (survival status, *p<*.05; N, *p*<.001; AJCC stage; *p*<.0001) except for T (**Figure 4B**). Of great interest, we noticed that the clinical features of COAD patients tended to be more severe as the risk scores increased, which reconfirmed the predictive value of the risk signature. Moreover, heat maps were also plotted to display the correlations between seven identified LMRGs and clinicopathological features in GSE40967 (**Figure 4C**) and TCGA-COAD (**Figure 4D**) data sets.

### Construction and assessment of a prognostic nomogram based on the risk score signature

**Figure 5:**
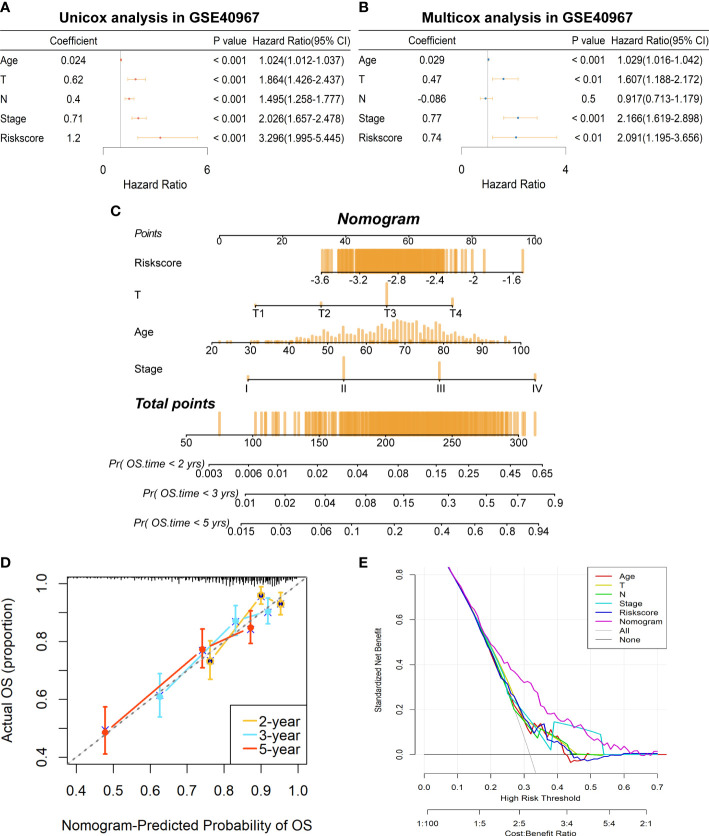
Establishment of a prognostic prediction nomogram based on the risk signature in GSE40967 training set. **(A)** The univariate Cox regression analysis of age, T, N, AJCC stage, and risk score for OS. **(B)** The multivariate Cox regression analysis of age, T, N, AJCC stage, and risk score for OS. **(C)** The prognostic nomogram was plotted to predict 2-, 3-, and 5-year survival probability of COAD patients. **(D)** The calibration plot was used to evaluate the prediction consistency of OS. **(E)** The DCA to assess clinical utility of the prognostic nomogram.

To further determine if the risk signature could serve as an independent prognostic indicator for COAD patients, we then conducted univariate and multivariate Cox regression analyses in GSE40967. The results disclosed that age, T, N, AJCC stage, and risk scores were all signally related to OS probability in the univariate Cox analysis (**Figure 5A**), whereas age, T, AJCC stage, and risk score remain independent prognostic factors after adjustment in the multivariate Cox analysis (**Figure 5B**). Subsequently, we developed a risk score–based nomogram to predict the individual OS probability of 2, 3, and 5 years according to the multivariate Cox analysis result (**Figure 5C**). Not surprisingly, the nomogram indicated that COAD patients with higher total points suffered a lower survival chance. Moreover, calibration of the nomogram was assessed in a calibration plot, which displayed great fitness through comparing observed to predicted risk (**Figure 5D**). The DCA curve of the nomogram presented a more favorable clinical net benefit than any single factor (**Figure 5E**). Up to this point, the prognostic nomogram based on LMRG-related risk signature has been verified to have better OS prediction capability for COAD patients.

### Evaluation of the correlation between risk score signature and immune landscape

**Figure 6:**
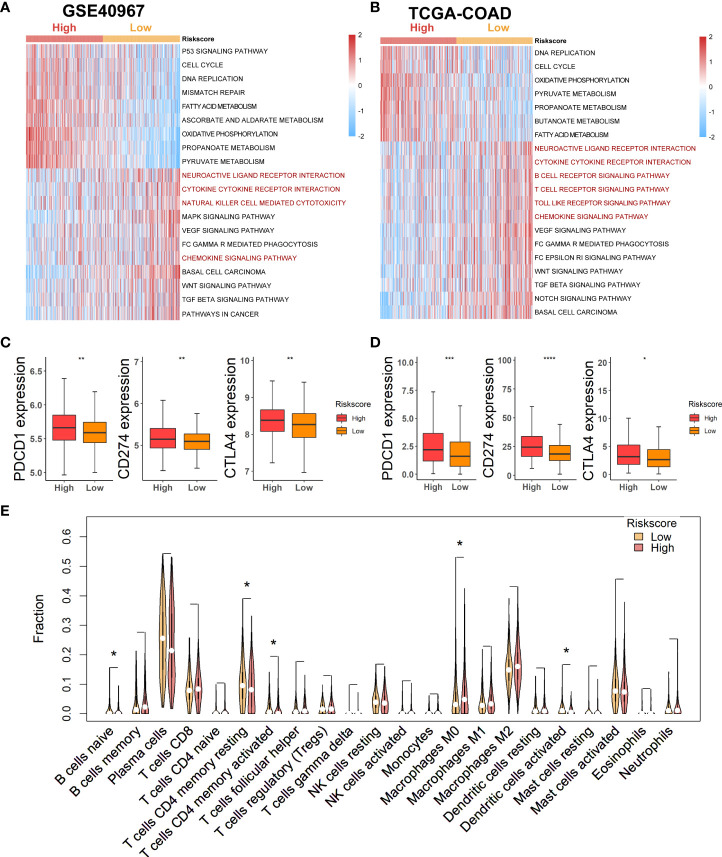
Estimation of potential relationship between various risk groups and immune landscape. **(A)** Results of GSVA analysis in GSE40967 to depict enriched immune-related pathways. **(B)** Results of GSVA analysis in TCGA-COAD to reveal enriched immune-related pathways. **(C, D)** Comparing expression levels of three classical markers of immune checkpoint between high- and low-risk groups in GSE40967 **(C)** and TCGA-COAD **(D)** separately. **(E)** Violin plots to show the relatively infiltrating abundances of 22 immune cells in COAD patients of GSE40967. *p < 0.05; **p < 0.01; ***p < 0.001; ****p < 0.0001.

As lactate metabolism has been recognized to play a vital role in TME, we first exploited “GSVA” enrichment analysis to unveil the discrepancy of immune-related pathways between high- and low-risk groups. As shown in **Figure 6**, neuroactive ligand receptor interaction, cytokine cytokine–receptor interaction, natural killer cell-mediated cytotoxicity, and chemokine signaling pathway were obviously enriched in the low-risk group of GSE40967 (**Figure 6A**), whereas neuroactive ligand receptor interaction, cytokine cytokine–receptor interaction, B-cell receptor signaling pathway, T-cell receptor signaling pathway, toll-like receptor signaling pathway, and chemokine signaling pathway were similarly enriched in the low-risk group of TCGA-COAD (**Figure 6B**). Then, we further compared the expression levels of common immune checkpoints within the two groups, and the results manifested that PD-1, PD-L1, and CTLA4 were all significantly augmented in the high-risk groups of both GSE40967 (**Figure 6C**) and TCGA-COAD data sets (**Figure 6D**), which unearthed the fact that COAD patients of high-risk groups may have an immunosuppressive TME and may also respond better to immunotherapy targeting the immune checkpoints.

To more robustly demonstrate the effect of various risk scores on TME, the CIBERSORT algorithm was performed to estimate the infiltrating degree of immune cells in the TME of COAD in GSE40967. From the result, we found that naive B cells, resting memory CD4+ T cells, activated memory CD4+ T cells, and activated dendritic cells were markedly enriched in the TME of low-risk group, whereas macrophages M0 were notably strengthened in the high-risk group (**Figure 6E**). Judging from the results above, COAD patients with low risk might attain an immune-activated TME, which also supported that low-risk patients had more favorable prognoses.

## Discussion

Although the treatment methods of COAD have been greatly improved, the prognoses of COAD patients remain dismal (30). Therefore, there is an urgent need to discover more accurate prognosis-related biomarkers and provide interventions as early as possible to improve the prognosis. Recently, it is worth drawing attention to the lactate metabolism, which has been reported to have played an unignorable role in tumorigenesis and progression, including COAD (10, 16). More strikingly, lactate was always produced by tumor cells and then accumulated in the TME, which subsequently generated some effects on tumor immune infiltrating cells, including inhibition of the functions of cytotoxic T, NK, and NKT cells (14, 15). Generally, the immunosuppressive role of lactate metabolism was associated with poorer response to immunotherapy of immune checkpoint inhibitors (ICIs). In several cancer types, high lactate dehydrogenase (LDH) levels have been verified to be independent biomarkers for predicting therapeutic response of ICIs (7, 8, 31). However, such research in COAD is still lacking. Based on the evidence above, we consolidated the mRNA expression profiles of LMRGs and the clinical data of COAD patients to explore novel prognosis-related biomarkers.

For searching the most effective LMRGs to construct the prognostic signature, we initially conducted univariate Cox regression analyses of OS in COAD patients of both GSE40967 and TCGA-COAD data sets. Furthermore, by optimization of eight available LMRGs with LASSO Cox regression analysis in GSE409678, seven pivotal genes were included to determine the risk signature, namely PLEC, BCS1L, CPT2, SDHB, COQ2, SLC39A8, and PDSS2.

Many studies have emerged implicating that PLEC (plectin) was a pro-tumorigenic regulator of tumor proliferation, migration, and invasion (32–34). Recent studies revealing the anticancer effect by directly targeting plectin have opened new avenues of research into plectin’s role in cancer (35–37). For CRC, IHC analyses demonstrate increased expression of plectin in COAD and locally invasive nests compared with normal tissues (38). Suppression of plectin inhibited adhesion, migration, and invasion of colon carcinoma cells (39). Strikingly, knockout of plectin has also been implicated to reduce the motility of dermal fibroblasts and T cells *in vitro* as well as impaired the infiltration of macrophages and T cells during wound healing *in vivo* (40). Our study indicates that high expression of PLEC is related to poorer OS and clinicopathological features in COAD, which is consistent with the above evidence. However, more mechanisms to underpin these observations remain to be elucidated.

The carnitine palmitoyltransferase (CPT), including CPT1 and CPT2, are identified as important mediators of fatty acid oxidation (FAO) (41). CPT2 promotes the β-oxidation of fatty acids (FAs) through facilitating the conversion of acetyl-coenzyme A (CoA) to fatty acyl-CoA (42). CPT2 silencing is reported to facilitate the tumor progression of hepatocellular carcinoma, which is reconfirmed by our results (43). In addition, FAO mediated by CPTs also played an important role in tumor immunity (44).

SDHB was one of the four subunits comprising the succinate dehydrogenase (SDH) enzyme complex, which is related to the tricarboxylic acid (TCA) cycle and oxidative phosphorylation (45). The lack of SDHB function promotes the occurrence and development of several cancers, including liver and pancreatic cancer (46, 47). Similarly, the result of our analyses indicate that high expression of SDHB was related with better prognosis in COAD.

PDSS2 (prenyldiphosphate synthase subunit 2) was characterized as a tumor suppressor, and introduction of PDSS2 into cancer cells has been verified to inhibit tumor growth (48, 49). Likewise, the tumor suppressive role of PDSS2 was further strengthened with findings of COAD presented in our study. Regrettably, cancer-related studies of the other few candidate genes, including BCS1L, COQ2, and SLC39A8, are still absent, and we will seriously consider deeper research in our future studies.

The above seven LMRGs were selected to establish the prognostic signature, and then, prognosis and clinicopathological relevance of the risk signature were comprehensively evaluated. Based on the survival analyses, COAD patients of the high-risk group were confirmed to have shorter time of OS, PFS, DFS, and DSS. Moreover, the AUC values of ROC curves displayed good accuracy of the risk model in predicting 1-, 2-, 3-, 4-, and 5-year OS probability in both data sets independently. Furthermore, COAD patients of both training and validation cohorts were divided into different subgroups according to survival status, T, N, and AJCC stage. Not surprisingly, we found that with the risk score increasing, COAD patients tended to have worse clinical outcomes. Specifically, progressively higher risk scores were related to larger tumor size, more metastatic axillary lymph nodes, and more severe AJCC stage. Even more remarkably, through univariate and multivariate Cox regression analyses, the risk signature was considered as an independent prognostic factor when adjusted with clinical variables containing age, T, N, and AJCC stage. In addition, development of a prognostic nomogram also unfolded good prediction consistency and potential clinical feasibility of the risk score in COAD patients. Nevertheless, larger cohorts are needed to confirm the above results in prospective studies.

We have already mentioned that lactate metabolism was verified to play a significant role in the TME, and advancements in research on the TME and immunotherapy are expected to provide more valid improvements for the prognoses of CRC patients (50). Above all, we explored the difference of immune-related pathways between high- and low-risk groups using the “GSVA” enrichment analysis, and we found that some common immune-related pathways were enriched in the low-risk group of two data sets, including neuroactive ligand receptor interaction, cytokine cytokine–receptor interaction, and chemokine signaling pathway. Meanwhile, by comparing the expression differences of immune checkpoints, PD-1, PD-L1, and CTLA4 were all significantly upregulated in the high-risk group. Moreover, the result of the CIBERSORT algorithm also demonstrated that naive B cells, resting memory CD4+ T cells, activated memory CD4+ T cells, and activated dendritic cells were enriched in the TME of the low-risk group. Clues from the above three aspects implied that COAD with a higher risk value might be more likely to be immunosuppressed, which was consistent with the consensus that activated lactate metabolism usually promoted immune invasion and suppressed antitumor immune responses. As for the response to immunotherapy, we assumed that high-risk COAD patients would be more sensitive to ICIs as targeting immune checkpoints will transform the immune microenvironment of COAD with high risk from immunosuppression relative to immunoactivation.

Though all the conclusions in the present study should be further verified by experimental data, it primarily provides a comprehensive description of LMRGs in COAD and constructed a ponderable risk signature, which also has novel implications for immunotherapy in COAD patients.

## Conclusion

In conclusion, our study establishes a reliable risk signature based on LMRGs for COAD patients. The signature was identified as an independent prognostic indicator through constructing a nomogram model that could accurately predict the survival probability of COAD patients. In addition, the potential relationship between different risk scores and tumor immune microenvironment was explored. Broadly speaking, our study may provide important preclinical implications for cancer research about lactate metabolism and COAD.

## Data availability statement

The original contributions presented in the study are included in the article/**Supplementary Material**. Further inquiries can be directed to the corresponding author.

## Ethics statement 

Ethical review and approval was not required for the study on human participants in accordance with the local legislation and institutional requirements. Written informed consent from the patients/participants or patients/participants’ legal guardian/next of kin was not required to participate in this study in accordance with the national legislation and the institutional requirements.

## Author contributions

All authors involved in the study have made a certain contribution, including designation (QX), data collection and analysis, manuscript drafting and revising (ZZ, YC, QL, XL, and QH) as well as study supervision (QX). All authors have read and approved the final manuscript submitted.

## Acknowledgments

We are very grateful for the data support provided by public databases and constructive and helpful comments from the reviewers.

## Conflict of interest

The authors declare that the research was conducted in the absence of any commercial or financial relationships that could be construed as a potential conflict of interest.

## Publisher’s note

All claims expressed in this article are solely those of the authors and do not necessarily represent those of their affiliated organizations, or those of the publisher, the editors and the reviewers. Any product that may be evaluated in this article, or claim that may be made by its manufacturer, is not guaranteed or endorsed by the publisher.
