# Supplementary material for: Establishment of lactate-metabolism-related signature to predict prognosis and immunotherapy response in patients with colon adenocarcinoma
Source: Front Oncol. 2022 Sep 14;12:958221. doi: 10.3389/fonc.2022.958221 (PMC9515658; doi:10.3389/fonc.2022.958221)
Supplement: Supplementary file 1 [file DataSheet_1.pdf]

Supplementary Material

SUPPLEMENTARY FIGURE LEGEND

Supplementary Figure 1: Identification of 228 available lactate metabolism-related genes. (A) Venn diagram to identify 228 overlapping LMRGs in TCGA-COAD and GSE40967 datasets. (B) The information of 228 candidate LMRGs.

A

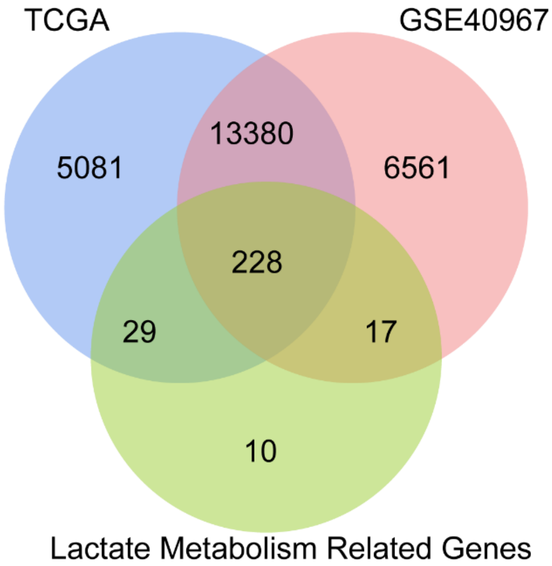

B

Supplement Table1. The information of 228 lactate metabolism-related genes								
1	COQ9	NDUFS4	POLG	PDP1	C1QBP	NDUFAF8	NDUFAF1	TUFM
2	ISCA1	FARS2	NDUFB3	COX16	EMB	SLC25A4	MVK	MTO1
3	HBB	NDUFA12	WARS2	SFXN4	SLC13A3	LYRM7	FBXL4	SDHA
4	AIFM1	MRPL12	NDUFAF6	CARS2	LDHA	COX6B1	PYGL	NUBPL
5	PIGA	SURF1	LARGE1	NGLY1	SIL1	PC	SLC25A19	ADAMTS13
6	FKRP	RARS2	SPP1	SLC19A1	DAG1	MIPEP	MRPS16	CHCHD10
7	PITRM1	SCO1	RNASEH1	CLPB	ACAT1	CYC1	TWNK	VPS13A
8	AARS2	NDUFAF2	TET2	TIMMDC1	TCIRG1	PET100	HTRA2	B3GALNT2
9	DNAJC19	NDUFS6	ECHS1	TIMM22	NDUFA13	UQCRQ	LIPT1	MYC
10	NDUFV2	HPDL	CHEK2	NDUFS3	RMND1	NAXE	POLG2	PHKG2
11	SLC16A7	ALDH4A1	EARS2	COX20	LRPPRC	CPT2	MRPL3	CYP27A1
12	RRM2B	TANGO2	LDHB	NDUFA2	BCS1L	FASTKD2	MDH2	INPP5K
13	NDUFB11	SLC39A8	SCO2	TP53	COQ4	TXN2	NDUFA6	NDUFAF3
14	MPC1	MTFMT	LYST	POMK	CD46	PNPLA8	ACAD9	DLD
15	TACO1	PDSS2	TRMU	IRAK1	PNPT1	LONP1	COX8A	POMT1
16	GOT2	NDUFB8	JAK2	FKTN	TK2	SLC7A7	TIMM50	POMGNT2
17	COX5A	MECP2	YARS2	GTPBP3	NDUFS2	SLC25A13	SUCLG1	GFM2
18	HIBCH	PLEC	SERAC1	TMEM126B	NDUFA9	COX4I1	TRMT10C	HSD17B10
19	NDUFS1	NDUFAF4	RXYLT1	OCRL	PLA2G6	MRPS28	PDSS1	SLC25A42
20	LIAS	TMEM70	NDUFV1	NDUFA1	PMPCB	NFS1	SLC25A10	PET117
21	SLC16A1	TRMT5	SQOR	MRPS34	SLC19A3	HS6ST2	MRPL44	CFI
22	NDUFA10	SLC25A3	COX15	DGUOK	COX10	ISCU	FOXRED1	FLI1
23	COG8	ACADM	SOD1	PDHX	NARS2	UQCRC2	DARS2	SDHB
24	STAT4	PUS1	UQCRB	AGK	NDUFA4	POMGNT1	PIEZO1	B4GAT1
25	ATAD3A	NDUFB10	HMGCL	USB1	SYNJ1	ATPAF2	PNPO	NDUFB9
26	PDHA1	OGDH	NDUFA8	GAA	UQCC3	RB1	MPV17	COL4A1
27	DNM1L	LDHD	COX14	MRPS14	SLC25A26	NDUFA11	CFH	CALR
28	GFM1	LIPA	MRPS22	TRAPPC12	ACAT2	NDUFAF5	POMT2	RPS14
29	KCNN4	NDUFS7	COQ2	PNPLA2				
